# Melatonin Enhances Crop Tolerance to Aluminum Toxicity in Acid Soils: A Comprehensive Review

**DOI:** 10.3390/plants15101465

**Published:** 2026-05-11

**Authors:** Muhammad Usman, Qing Li, Xinqi Peng, Yongxiu Xing, Muhammad Farooq, Dengfeng Dong

**Affiliations:** 1 Guangxi Key Laboratory of Agro-Environment and Agric-Products Safety, College of Agriculture, Guangxi University, Nanning 530004, China; usmankhan6191@gmail.com (M.U.);; 2 State Key Laboratory for Conservation and Utilization of Subtropical Agro-Bioresources, College of Life Science and Technology, Guangxi University, Nanning 530004, China

**Keywords:** melatonin, Al^3+^ toxicity, soil acidity, oxidative stress, root meristem, antioxidant defense, abiotic stress tolerance, sustainable agriculture

## Abstract

Soil acidity is a major constraint in many agricultural regions, where increased aluminum (Al^3+^) solubility at low pH severely affects plant health by inhibiting root elongation, disrupting nutrient uptake, and inducing oxidative stress. Recent studies have highlighted melatonin, a widely occurring indoleamine with strong antioxidant and stress-modulating properties, which alleviates Al-induced damage in crops. This review synthesizes current physiological, biochemical, and agronomic evidence demonstrating that exogenous melatonin enhances plant tolerance to aluminum toxicity. Across multiple model and crop species, melatonin application has been shown to improve root elongation by 20–45%, reduce lipid peroxidation by 30–60%, and enhance key antioxidant enzymes such as SOD, POD, and CAT by 25–70% under Al stress. Case studies in soybean, wheat, maize, and rice further indicate that melatonin protects root meristems from oxidative damage, stabilizes photosynthetic machinery, and improves nutrient acquisition. In acidic soils (pH 4.5), melatonin-treated soybean exhibited 28% greater biomass and 15–22% higher N and P uptake, while wheat plants demonstrated 10–18% higher grain filling under field-simulated Al stress. Emerging long-term studies show that melatonin also benefits soil health. Multi season experiments reveal that melatonin enhances root exudates that support beneficial rhizosphere microbes, increases soil enzymatic activities (urease, phosphatase) by 20–35%, and lowers exchangeable Al by 12–18%. These improvements contribute to cumulative yield gains of 10–18% over successive cropping cycles. Additionally, genetic approaches aimed at increasing endogenous melatonin levels in plants have demonstrated 12–30% yield improvement in acid soil conditions. This review highlights the need for multi-year, multi-location studies to further clarify how melatonin can support sustainable agricultural practices, enhance soil fertility, and mitigate aluminum toxicity in acid-affected regions.

## 1. Introduction

Soil acidity is a major global constraint in agriculture, significantly reducing crop productivity and soil health. Acidic soils (pH < 5.5) limit plant growth by causing nutrient deficiencies, restricting root development, and lowering microbial activity [1]. One of the most critical consequences of low soil pH is the increased solubility of aluminum (Al^3+^), which is highly toxic to plants. Elevated Al levels inhibit root elongation and impair root function, ultimately reducing crop yields and threatening food security, particularly in regions naturally affected by acidity, acid rain, or intensive agricultural practices [2].

Aluminum toxicity is therefore recognized as a major limitation in acid soils worldwide. When soil pH drops below 5.0, soluble Al forms disrupt plant physiological and biochemical processes, including carbohydrate, protein, and lipid metabolism, leading to reduced root growth and crop productivity [3]. Given that nearly 30% of the Earth’s land surface and about half of the world’s potentially arable land are acidic, Al^3+^ toxicity represents a significant global agricultural challenge [4].

Recent studies have highlighted the potential of melatonin, a naturally occurring indoleamine, to alleviate the adverse effects of aluminum under acidic soil conditions [5]. In plants, melatonin functions as a potent antioxidant, scavenging reactive oxygen species (ROS) and reducing oxidative damage, while also modulating growth, development, and stress-responsive signaling pathways [6].

Since its identification in plants (Pharbitis nil) in 1993 [7] It has been shown to regulate key physiological processes such as root development, seed germination, photosynthesis, and senescence. Both endogenous and exogenous melatonin enhance tolerance to a wide range of abiotic stresses, including salinity, temperature extremes, nutrient deficiency, and heavy metal toxicity [8,9]. Under metal stress conditions, melatonin improves plant tolerance by enhancing antioxidant enzyme activity, promoting sequestration of toxic ions into vacuoles, and regulating redox homeostasis and signaling molecules such as H_2_O_2_ and nitric oxide (NO) [10]. Despite these advances, the specific mechanisms by which melatonin alleviates aluminum toxicity remain incompletely understood [11].

Previous studies further indicate that melatonin enhances plant tolerance to aluminum and other abiotic stresses by strengthening antioxidant defenses, stabilizing cellular structures, and maintaining redox homeostasis [12]. Its application may therefore offer a sustainable and environmentally friendly strategy for improving crop performance under acidic soil conditions [13].

Therefore, this review aims to provide a comprehensive overview of the potential role of melatonin in mitigating aluminum-induced soil acidity and enhancing plant tolerance. It synthesizes current knowledge on melatonin-mediated protective mechanisms, highlights its possible agricultural applications, and identifies key research gaps that must be addressed to optimize its use in sustainable crop production systems.

### 1.1. Soil Acidity and Aluminum Toxicity

Soil acidity arises from both natural processes and human activities, leading to reduced soil pH and significant agricultural challenges. Naturally, acidity can develop through the weathering of acidic parent materials such as granite and sandstone, as well as the leaching of basic cations, including calcium, magnesium, potassium, and sodium, which are subsequently replaced by more acidic ions such as hydrogen and aluminum. In regions with high rainfall, soil acidity is further intensified because slightly acidic rainwater, containing dissolved CO_2_, accelerates the leaching of base cations [14].

Human activities also contribute significantly to soil acidification, particularly through the use of nitrogenous fertilizers such as ammonium sulfate and urea. During nitrification, ammonium is converted into nitrate by soil microorganisms, a process that releases hydrogen ions and lowers soil pH [15]. In addition, industrial pollution, especially emissions of sulfur dioxide and nitrogen oxides, leads to acid rain, which further accelerates soil acidification. Continuous cropping and the removal of crop residues can also reduce the soil’s buffering capacity, thereby intensifying acidification over time [16].

More than 40% of the world’s arable soils are acidic and therefore susceptible to the harmful effects of aluminum (Al^3+^), which is considered a major factor limiting the fertility of most acid soils and restricting plant growth, particularly in kaolinitic soils at pH below 5.0–5.5 [17]. High concentrations of soluble Al^3+^ are widely recognized for their detrimental effects on plant growth; however, the mere presence of Al in the solid phase or soil solution does not always result in toxicity symptoms. Additional factors including soil pH, formation of insoluble precipitates, protection by competing ions, and ionic strength also influence Al toxicity. Among these, soil pH and the specific chemical forms of Al^3+^ in the soil solution are considered more reliable indicators of toxicity than exchangeable Al, its saturation, or total Al^3+^ activity [18]. Only monomeric forms of aluminum are regarded as highly toxic, particularly Al^3+^ and monomeric aluminum hydroxyl species, whereas soluble organo-Al, Al–F, and Al–SO_4_ complexes are comparatively less phytotoxic.

Soil acidity alters nutrient availability and can severely affect plant health as well as the physical and chemical properties of the soil; among its most harmful consequences is aluminum toxicity [19]. Under acidic conditions, aluminum that normally exists in non-toxic forms becomes solubilized as Al^3+^, a form that is highly toxic to plants. This soluble aluminum inhibits root growth by binding to root tips and disrupting cell division and elongation. As a result, plants develop shallow and poorly functioning root systems that limit water and nutrient uptake, ultimately leading to reduced plant growth and lower crop productivity [20].

Toxic concentrations of aluminum also negatively affect the physical and chemical properties of soil and the activity of beneficial microorganisms. Aluminum can precipitate soil colloids, thereby disrupting soil aggregation, porosity, and water infiltration [21]. These changes may increase the risk of waterlogging and soil erosion in agricultural fields. Furthermore, excessive aluminum can interfere with mycorrhizal fungi and nitrogen-fixing bacteria, key components of the plant–soil ecosystem, leading to reduced soil fertility and a diminished capacity of plants to survive and grow [22].

**Figure 1:**
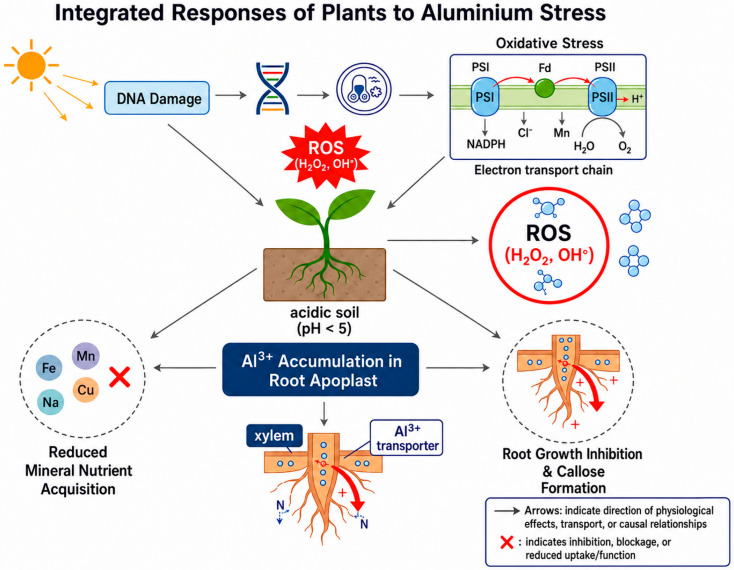
Responses of plants to Al^3+^ toxicity in soil.

Aluminum solubility in soil is closely controlled by soil pH. At pH values of 5.5 or higher, aluminum remains in insoluble, non-toxic forms that are generally harmless to plants. As soil pH drops below 5.5, aluminum becomes increasingly soluble, raising the concentration of toxic Al^3+^ in the soil solution. Because of this inverse relationship, even small decreases in pH can substantially increase aluminum availability. When soil pH reaches approximately 4.5 or lower, aluminum solubility often rises to levels that are toxic to most plants [23]. Figure 1, given below, indicates plant responses to aluminum toxicity in soil.

Soil pH not only regulates aluminum solubility but is also influenced by dissolved aluminum, creating a feedback mechanism that can further increase soil acidity. As aluminum becomes more soluble, it displaces basic cations from soil particles, which accelerates the decline in soil pH and enhances aluminum availability [24]. This process restricts plant growth by reducing nutrient availability and impairing root development under highly acidic conditions. Therefore, managing soil acidity and aluminum toxicity is essential for maintaining soil health and ensuring sustainable agricultural production [25].

### 1.2. Mechanisms of Aluminum Toxicity

Plant uptake of aluminum (Al^3+^) increases when soil pH falls below 5.5 due to the solubilization of toxic Al^3+^ ions. These ions can enter plant roots through two main pathways: the apoplastic route, where Al^3+^ moves through cell wall spaces without crossing the plasma membrane, and the symplastic route, where Al^3+^ is transported across the plasma membrane into the cytoplasm of root cells [26].

The root apex, which contains young, actively dividing cells, is the primary site of aluminum adsorption. Its large surface area and abundance of negatively charged binding sites in the cell walls promote the attachment of positively charged Al^3+^ ions. After entering root tissues, aluminum can accumulate in the cell wall, cytoplasm, and vacuoles, where it exerts toxic effects on cellular function [27]. Melatonin has been shown to mitigate early aluminum uptake by strengthening cell wall integrity and altering pectin composition, thereby reducing Al^3+^ binding to negatively charged sites and limiting its entry into root cells. Aluminum accumulation in plant tissues induces a range of harmful physiological and biochemical changes that impair plant growth and development. One of the earliest and most severe targets of aluminum toxicity is the cell wall, where aluminum interacts with pectin, increasing wall rigidity and restricting root elongation [28]. Melatonin counteracts these effects by regulating cell wall-modifying enzymes, such as pectin methylesterases and expansins, thereby maintaining cell wall flexibility and promoting root growth.

Within the cytoplasm, aluminum disrupts several critical biochemical processes. It can inhibit enzyme activities, particularly those involved in phosphate transport, by binding phosphate groups and rendering them unavailable for cellular use. This interference reduces ATP production and impairs energy metabolism in plant cells [29]. Melatonin protects these metabolic processes by stabilizing enzymes, maintaining mitochondrial function, and sustaining ATP production under aluminum stress. Additionally, aluminum induces oxidative stress by promoting the generation of reactive oxygen species (ROS), which damage cell membranes, proteins, and DNA. Although plants activate antioxidant defenses in response to ROS, excessive aluminum can overwhelm these systems, leading to cellular injury and programmed cell death. The effects of aluminum toxicity and the protective role of melatonin are presented in Table 1.

**Figure 2:**
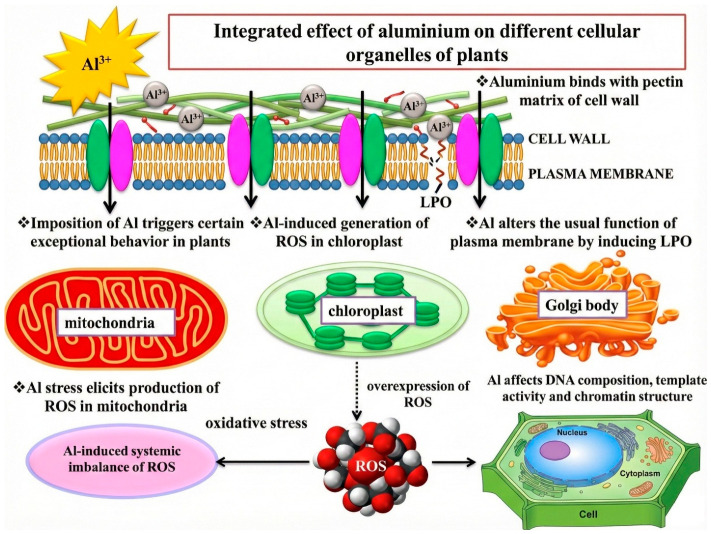
Toxicity of aluminum on different organelles of plants.

Melatonin functions as a powerful antioxidant by directly scavenging reactive oxygen species (ROS) and upregulating antioxidant enzymes, including SOD, CAT, and APX, thereby protecting cellular structures from oxidative damage. Aluminum also disrupts the cytoskeleton, affecting microtubules and actin filaments that are essential for cell shape, intracellular transport, and division (Figure 2). This disruption interferes with cytokinesis and cell elongation, resulting in reduced root and shoot growth [20]. Melatonin mitigates these effects by stabilizing the cytoskeleton under stress, maintaining structural integrity, and supporting normal cell division and elongation.

High concentrations of aluminum severely impair root development, which is one of the most dramatic effects of aluminum toxicity in plants. The root apex, containing young and actively dividing cells, is particularly vulnerable. Aluminum binds to the cell walls of root tips, restricting cell wall expansion and elongation, while also interfering with cell division in the root meristem, resulting in reduced root length and surface area [32]. Melatonin mitigates these effects by promoting root meristem activity, restoring auxin transport, and enhancing both root elongation and lateral root formation under aluminum stress. Impaired root growth also limits water and nutrient uptake. Aluminum competes with essential cations such as phosphorus, potassium, calcium, and magnesium for binding sites on root cell walls and transporters, leading to nutrient deficiencies and further stress, which slows overall plant growth and productivity [18]. Melatonin helps maintain nutrient homeostasis by regulating ion transporters, improving the uptake of essential nutrients, and counteracting aluminum-induced nutrient deficiencies.

Aluminum disrupts transport and signaling processes within the plant, leading to alterations in hormone levels, particularly auxins, which are essential for root development [24]. By impairing auxin transport, aluminum stress can result in distorted root morphology, reduced lateral root formation, and overall poorly developed root systems [33].

Melatonin helps maintain hormonal balance and ensures proper auxin distribution, thereby promoting healthier root architecture. In conclusion, aluminum toxicity affects plants in multiple ways, including impaired root growth and function, disruption of key physiological and biochemical processes, and interference with nutrient uptake. These combined effects compromise plant health and reduce agricultural productivity, highlighting the need for effective strategies to mitigate aluminum toxicity in acidic soils. The application of melatonin addresses these multi-level effects by enhancing antioxidant defenses, stabilizing cell walls and the cytoskeleton, maintaining energy metabolism, regulating nutrient and hormone transport, and supporting root development. Collectively, these protective mechanisms make melatonin a promising agent for improving plant resilience in acidic soils and alleviating aluminum toxicity [34].

### 1.3. Melatonin: Properties and Functions

Melatonin (N-acetyl-5-methoxytryptamine) is an indole tryptamine first identified in the pineal gland of cows and initially characterized based on its chemical structure [35]. In animals, melatonin exerts a variety of physiological effects, including regulation of sleep, delay of aging processes, suppression of allergic responses, and modulation of the immune system. Due to these diverse functions, melatonin is widely used as an active ingredient in medicines and health-care products for the regulation of circadian rhythms and sleep [36,37].

The melatonin content in plants varies depending on the species, growth stage, and environmental conditions, including climate. Additionally, differences in extraction and detection methods can influence the measured melatonin levels in plant samples [38]. Unlike animals, which can move to avoid stress, plants are immobile and must rely on internal mechanisms to cope with adverse environments. High levels of melatonin, which functions as an antioxidant, have been observed in plants growing in regions with intense ultraviolet radiation, such as the Mediterranean and the Alps, helping to reduce oxidative damage [39].

Melatonin exhibits strong free radical scavenging activity and can also enhance the synthesis of other antioxidant enzymes, such as superoxide dismutase [40]. In addition, it contributes to fruit quality and preservation by promoting ripening and delaying senescence. At the molecular level, it regulates genes involved in secondary metabolism, stress responses, ethylene (ET) synthesis and signaling, flavonoid production, cell wall composition, carbohydrate metabolism, and the ascorbate glutathione (ASA–GSH) cycle. Melatonin can modulate ET-related transduction genes (EIL1/3 and ERF2) and biosynthesis genes (ACS and ACO), either upregulating or downregulating their expression [41]. Growth effects of melatonin on different plant species are given in Table 2.

The mechanisms by which melatonin alleviates stress in plant seeds are becoming increasingly clear. Melatonin helps maintain seed viability and vigor due to its strong antioxidant activity. Melatonin also regulates hormone-related genes, enhancing gibberellin levels (particularly GA_4_) through GA20ox and GA3ox genes and modulating ABA metabolism via ABA 8′-hydroxylase and NCED2 genes, maintaining the balance of GA and ABA during germination [40]. Under stress, plants activate antioxidant enzymes such as superoxide dismutase (SOD), catalase (CAT), and peroxidase (POD), while upregulating their corresponding genes (Cu-Zn SOD, Fe-Zn SOD, CAT, POD), allowing melatonin to scavenge reactive oxygen species, including hydrogen peroxide. Studies have shown that melatonin improves germination rates in various species (Limonium bicolor seeds and Cucumis melo seeds by optimizing soluble sugar utilization, promoting protein synthesis, and increasing amylase and α-amylase activities.

Overall, melatonin acts as a key antioxidant and stress-response regulator in plants. It directly scavenges reactive oxygen and nitrogen species, enhances the activities of antioxidant enzymes, and upregulates stress-responsive genes, including Cu/Zn-SOD, CAT, APX1/2, and POD. These coordinated responses protect lipids, proteins, and nucleic acids from oxidative damage. Melatonin has been shown to mitigate oxidative stress under a variety of adverse conditions, particularly aluminum toxicity [52]. In most studies, effective concentrations for foliar or root application range from 50 to 200 µM, with approximately 100 µM being the most common dose for reducing aluminum-induced ROS in crops such as rice, wheat, soybean, and maize.

**Figure 3:**
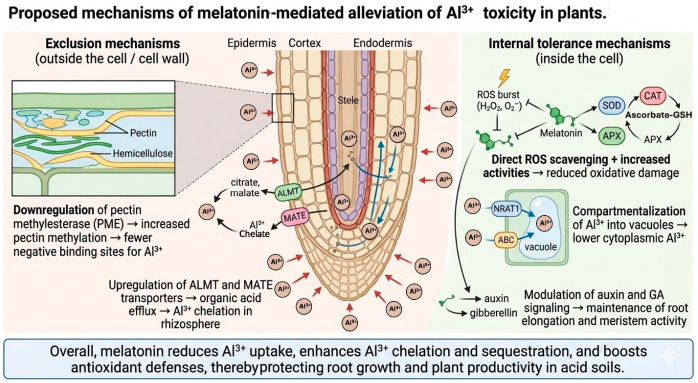
Proposed mechanisms of melatonin-mediated alleviation of Al^3+^ toxicity in plants.

Melatonin inhibits oxidative stress in a number of ways, which helps plants resist abiotic stresses [53]. Exogenous application increases endogenous melatonin levels in wheat by upregulating *TaSNAT* transcripts, which encode key enzymes in the MT biosynthesis pathway [54]. Melatonin also upregulates antioxidant-related genes; for instance, it enhances the expression of *APX1/2*, CAT1, and FSD1, thereby increasing the activities of APX, CAT, and SOD in *Arabidopsis* [30]. In addition, melatonin elevates the expression of genes involved in ascorbate metabolism, such as VTC4 and APX4, further improving the plant’s antioxidant capacity [55]. The coordinated plant-level mechanisms through which melatonin alleviates Al^3+^ toxicity including cell wall modification, organic acid exudation, antioxidant defense, vacuolar sequestration, and hormonal regulation are summarized schematically in Figure 3.

Melatonin is a highly conserved and ubiquitous molecule involved in diverse physiological processes in both plants and animals. In animals, it is best known for regulating circadian rhythms and sleep–wake cycles by acting as an internal signal of day–night timing in response to external light–dark conditions. Beyond circadian regulation, melatonin also participates in reproductive functions, immune responses, and seasonal physiological changes [41].

Melatonin is well recognized for its multiple roles in plant growth, development, and stress tolerance. It functions as a growth-promoting regulator involved in seed germination, root development, and flowering. In addition, melatonin regulates the expression of genes associated with plant defense against both biotic and abiotic stresses. It also interacts with other plant hormones, including auxins, gibberellins, and cytokinins, thereby enhancing its regulatory effects on plant growth and stress responses [56].

Among the diverse functions of melatonin, its strong antioxidant capacity is particularly significant and is considered a primary role of this indole compound. Melatonin acts as a direct free radical scavenger, reacting with reactive oxygen and nitrogen species to eliminate highly toxic radicals that can cause cellular damage (Table 3). It also enhances the activity of antioxidant enzymes, including superoxide dismutase (SOD), catalase, and glutathione peroxidase, thereby strengthening the cellular defense system against oxidative stress [57]. This potent antioxidant function makes melatonin crucial for protecting plants from aluminum (Al^3+^) toxicity, as well as other heavy metal stresses. Consequently, melatonin helps maintain cellular homeostasis and preserves essential cellular components lipids, proteins, and nucleic acids from oxidative damage and peroxidation [58].

### 1.4. Abiotic Stress Tolerance Mechanisms of Melatonin

Melatonin is widely recognized for its capacity to enhance plant tolerance to diverse abiotic stresses [9,63,64]. Melatonin protects plants through interconnected mechanisms, primarily by directly scavenging reactive oxygen and nitrogen species (H_2_O_2_, O_2_^−^, OH•, and NO), with its metabolites further sustaining antioxidant protection [40,57,58,65]. Melatonin enhances endogenous antioxidant defenses by upregulating key enzymes (SOD, CAT, POD, and APX) and activating the ascorbate-glutathione cycle [5,8,55,59]. Melatonin restores cellular homeostasis by stabilizing membranes, reducing lipid peroxidation, regulating ion balance, and preserving organelle integrity [53,66]. It also modulates phytohormone signaling networks, including auxin, gibberellin, abscisic acid, cytokinin, and ethylene, to regulate stress adaptation [33,56,67]. Finally, melatonin regulates stress-responsive genes, including those involved in protein protection and metal detoxification [7,9,68]. These effects are dose-dependent, with low to moderate concentrations enhancing stress tolerance, while higher levels may inhibit growth, and optimal doses vary among species [5,13,45,48].

### 1.5. Melatonin-Mediated Al^3+^ Toxicity Mitigation: Plant-Level Mechanisms

Melatonin alleviates Al^3+^ toxicity through six coordinated plant-level mechanisms that target the principal sites of Al-induced injury: root cell wall integrity, oxidative metabolism, ion balance, vacuolar detoxification, hormone regulation, and cytoskeletal stability based on evidence from hydroponic, pot, and field studies across major crops. These responses involve both direct biochemical protection and indirect gene-regulatory effects and are partially distinct from general heavy-metal tolerance pathways.

Melatonin limits Al^3+^ entry primarily by altering root cell-wall composition and membrane transport processes [10,69]. Downregulation of pectin methylesterase increases pectin methylation and reduces negatively charged Al binding sites, while enhanced hemicellulose and cellulose synthesis strengthen the apoplastic barrier and decrease Al penetration [70,71,72]. Aluminum tolerance in crop genotypes primarily depends on external Al exclusion via ALMT and MATE-mediated organic acid efflux from root apices, alongside internal mechanisms that maintain plasma membrane integrity and H^+^-ATPase activity to limit symplastic Al accumulation [73,74,75].

Melatonin mitigates Al^3+^ induced oxidative stress through direct scavenging of reactive oxygen and nitrogen species and stimulation of antioxidant defense systems [5,7,12]. It reduces lipid peroxidation and ROS accumulation, enhances antioxidant enzyme activities (SOD, CAT, POD, APX), and strengthens the ascorbate–glutathione cycle, with particularly strong protection in the root apex where Al toxicity is most pronounced [55,76,77]. Melatonin promotes internal detoxification primarily through enhanced vacuolar sequestration of Al^3+^ and activation of antioxidant defense systems that protect cellular compartments [10,31]. This substantially lowers cytoplasmic Al^3+^ concentrations and complements external exclusion mechanisms, particularly in crop species where internal tolerance strategies predominate [73,78].

**Figure 4:**
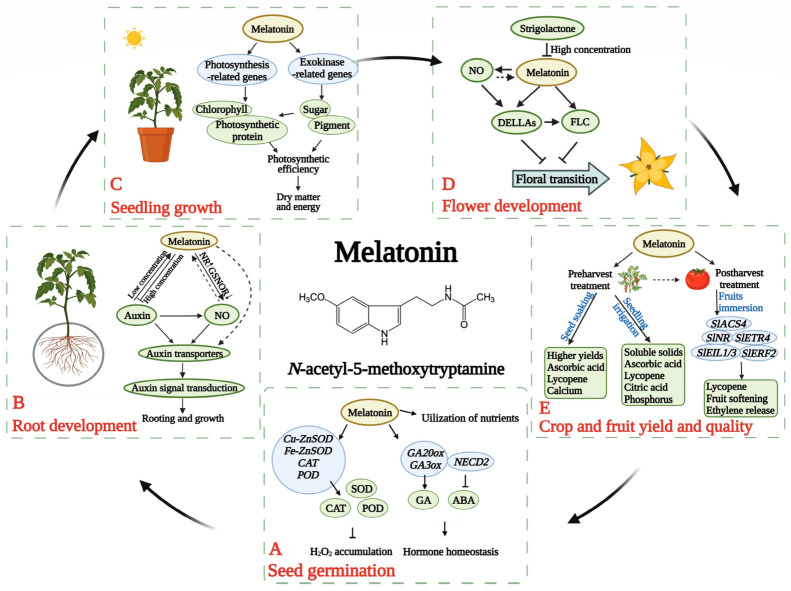
Proposed mechanisms by which melatonin regulates plant growth and development.

Al^3+^ toxicity disrupts the uptake of essential nutrients and inhibits root growth, leading to compromised physiological performance in acidic soils [32]. Exogenous application of melatonin has been shown to alleviate Al-induced stress by enhancing antioxidant defenses, reducing Al accumulation in roots, and improving overall plant biomass and root elongation [13,79]. Transcriptomic and physiological analyses further indicate that melatonin can modulate ion homeostasis and reduce reactive oxygen species under Al^3+^ stress [80]. Collectively, these studies demonstrate that melatonin enhances plant tolerance to Al^3+^ toxicity through multiple complementary mechanisms, including improved oxidative stress mitigation and maintenance of cellular homeostasis. Coordinated physiological adjustments induced by melatonin contribute to enhanced root growth and recovery under aluminum stress. Exogenous melatonin has been shown to mitigate aluminum-induced root growth inhibition by modulating signaling pathways associated with cell division and quiescent center activity, improving root elongation in Arabidopsis [81]. In cereal crops like maize, melatonin application alleviates growth inhibition, reestablishes redox homeostasis, and improves overall biomass under aluminum stress [76]. Melatonin also reduces oxidative damage and modulates antioxidant enzyme activities, contributing to the maintenance of root cellular integrity and structural resilience under Al^3+^ exposure [10]. Collectively, these mechanisms help explain melatonin’s capacity to sustain plant growth and physiological performance in acidic soil. Different mechanisms by which melatonin controls the growth of plants are shown in Figure 4. Unlike conventional soil amendments such as lime, melatonin does not directly modify bulk soil chemistry or Al^3+^ solubility; instead, it indirectly improves rhizosphere conditions through plant-mediated processes, particularly by enhancing root exudation and stimulating beneficial microbial interactions. Evidence from several studies demonstrates that these rhizosphere-level responses reduce Al^3+^ bioavailability, enhance nutrient cycling, and contribute to long-term soil health, creating a sustainable plant–soil feedback system.

The principal mechanism involves increased secretion of organic acids, including citric, malic, oxalic, and succinic acids, which chelate Al^3+^ in the rhizosphere, forming non-toxic complexes and limiting its uptake by roots [5,13]. Melatonin amplifies this response by upregulating genes involved in organic acid synthesis, such as citrate synthase and malate dehydrogenase, and by enhancing the expression of membrane transporters responsible for organic acid efflux, including ALMT and MATE family proteins [10]. These responses are species dependent, with monocots primarily exuding malate and citrate, and dicots favoring citrate and oxalate, helping explain variation in melatonin-mediated Al^3+^ tolerance among crop groups. Collectively, the evidence indicates that melatonin alleviates aluminum toxicity predominantly by reshaping rhizosphere biochemical dynamics rather than directly altering soil chemical properties. The available experimental evidence supporting each proposed mechanism of melatonin-mediated alleviation of Al^3+^ toxicity, including the crop species, experimental system, Al^3+^ concentration, melatonin dose and application, key response parameters, direct validation under Al^3+^ stress, and strength of evidence, is summarized in Table 4.

### 1.6. Modulation of Rhizosphere Microbial Communities

Melatonin contributes to aluminum (Al^+3^) tolerance not only through plant physiological regulation but also by influencing rhizosphere microbial dynamics. Although direct evidence under Al^3+^ stress remains limited, melatonin has been shown to alter the composition of soil microbial communities under other abiotic stresses such as drought, increasing the relative abundance of certain beneficial taxa and modifying rhizosphere metabolite profiles that can indirectly support microbial recruitment and plant stress resilience [86,87]. In some plant systems, exogenous melatonin significantly modified rhizosphere bacterial and fungal community structure under stress, suggesting that melatonin-mediated changes in root carbon allocation and soil metabolites may influence microbial assemblages [88]. Furthermore, melatonin has been shown to enhance *Arbuscular mycorrhizal* fungal colonization in several plant species under environmental stresses, indicating a potential role for melatonin in strengthening symbiotic plant –microbe interactions, though this has not yet been demonstrated specifically under Al^3+^ toxicity [89].

Plant growth-promoting rhizobacteria (PGPR), including genera such as *Bacillus*, *Pseudomonas*, and *Azospirillum*, as well as nitrogen-fixing rhizobia and *Azotobacter*, are well established components of beneficial rhizosphere communities that enhance nutrient availability, organic acid release, and phytohormone production, thereby supporting stress tolerance [90]. While melatonin’s ability to recruit these specific taxa under aluminum stress has not been directly shown, this conceptual framework provides a basis for future research into microbiome-mediated mitigation of Al^3+^ toxicity. Changes in rhizosphere microbial activity can also influence soil enzyme functions such as urease and phosphatase activities, which are important for nitrogen and phosphorus cycling, further contributing to soil health. Although variation in soil texture and organic matter content can modulate microbial abundance and responsiveness, the interplay between melatonin application, microbial community dynamics, and soil physicochemistry under Al^3+^ stress remains an active area for investigation.

### 1.7. Melatonin-Mediated Aluminum Detoxification and Rhizosphere pH Regulation

Although melatonin is widely reported as a plant stress regulator, the hypothesis that melatonin directly chelates Al^3+^ in the rhizosphere to form stable, non-toxic complexes lacks robust biochemical evidence under realistic soil conditions. Lower stability constants for melatonin metal complexes compared with classic organic acid chelators such as citrate and malate are suggested by indirect comparisons in the broader metal binding literature, indicating that any direct Al^3+^ binding by melatonin is weak and unlikely to be a major detoxification pathway in soil environments where organic acids and soil organic matter are abundant. In contrast, consistent in vivo evidence indicates that melatonin enhances plant aluminum tolerance primarily by stimulating organic acid anion exudation from roots. For example, melatonin application increased citrate and malate release from soybean roots under Al^+3^ stress, which enhances Al^3+^ chelation in the rhizosphere and reduces its bioavailability. Similarly, melatonin improved aluminum tolerance in alfalfa through physiological and transcriptomic alterations associated with stress responses, including enhanced antioxidant defenses and modulation of root exudation patterns [5].

Melatonin does not directly alter bulk soil pH but may influence proton dynamics at the root–soil interface via indirect physiological effects. Exogenous melatonin has been associated with modulation of ion transport and membrane stability in other abiotic stress contexts, including salinity, where melatonin aids maintenance of plasma membrane H^+^-ATPase activity and ionic homeostasis; such effects could modestly influence rhizosphere proton gradients and local pH microenvironments around root surfaces [91]. However, these responses are context-dependent and generally limited in magnitude. Collectively, available evidence supports a model in which melatonin’s mitigation of aluminum toxicity arises mainly from plant-mediated processes, such as the enhancement of organic acid exudation that binds Al^3+^ outside the root, upregulation of internal antioxidant defenses, and modulation of ion transport pathways rather than direct chelation of Al^3+^ or broad changes in soil chemistry. This perspective underscores the need for future research to clarify the molecular mechanisms by which melatonin influences rhizosphere pH dynamics and organic acid exudation under aluminum stress.

### 1.8. Case Studies and Experimental Evidence

Several studies have investigated the role of melatonin in mitigating aluminum (Al^3+^) toxicity in plants, and the available evidence is highly consistent [7,12]. Research has shown that melatonin application enhances plant tolerance to Al^3+^ stress by improving growth performance, physiological function, and biochemical status [92,93]. It has been shown that the enhancement of melatonin application can improve the plant tolerance to aluminum stress in terms of growth index, physiological potential as well as biochemical status. For instance, studies conducted on rice, wheat, and maize demonstrated that melatonin treatment promotes root growth, increases chlorophyll content, and enhances photosynthetic activity under Al stress [94,95,96]. These results collectively indicate protection of key physiological processes rather than isolated trait improvements. A compilation of representative experimental studies investigating the effects of melatonin on plant responses to aluminum (Al^3+^) toxicity, including details on crop species, experimental conditions, Al^3+^ stress levels, melatonin application protocols, observed phenotypic responses, and proposed mechanisms, is presented in Table 5.

Exogenous melatonin was applied to aluminum-stressed wheat plants, resulting in a significant reduction in oxidative stress indicators, including lower malondialdehyde (MDA) levels and increased activities of antioxidant enzymes such as superoxide dismutase (SOD) and catalase [7]. These findings demonstrate that melatonin-regulated antioxidant defense mechanisms play a crucial role in protecting plants from the detrimental effects of aluminum toxicity. Across different studies, experimental conditions have varied in terms of melatonin concentration, treatment duration, and application method, including foliar spraying and root drenching [12,76]. Despite these methodological differences, a consistent pattern has emerged: melatonin reduces aluminum uptake and enhances plant resilience. For instance, hydroponic experiments with soybean plants showed that melatonin-treated plants accumulated significantly less aluminum in root tissues than untreated controls. This reduction was accompanied by fewer symptoms of aluminum toxicity, such as root tip browning and inhibited growth [97]. Some controlled experiments have also used soil-based systems to better simulate field conditions. In these studies, melatonin application improved soil pH stability and reduced levels of soluble aluminum, thereby decreasing its toxicity to plant roots [98]. Furthermore, field trials have demonstrated that melatonin treatment in crops such as maize not only alleviates aluminum stress but also increases overall yield, highlighting its potential relevance for practical agricultural applications [99].

Evidence for melatonin’s ability to alleviate aluminum stress varies according to the physiological and biochemical responses of different plant species. Studies in rice and barley indicate that melatonin strongly enhances root growth and nutrient uptake under aluminum stress, whereas tomato and cucumber show moderately high responsiveness. These interspecific differences may arise from variation in melatonin receptor expression, antioxidant enzyme activity, and the inherent stress tolerance capacity of each species [6,100]. In dicotyledonous plants such as tomato and soybean, melatonin primarily promotes root development and suppresses aluminum-induced oxidative damage. In contrast, monocotyledonous plants, including rice and wheat, tend to exhibit greater improvements in photosynthetic efficiency and chlorophyll content when treated with melatonin under aluminum stress [101,102].

Despite these encouraging findings, inconsistencies persist among studies. Variations in melatonin concentration, soil type, and application method often produce differing plant responses, and doses that are effective for one species may not be suitable for another. Hydroponic experiments generally report stronger improvements than soil-based systems, likely due to more uniform melatonin availability and uptake. In addition, long-term field data remain limited, highlighting the need to validate laboratory results under diverse environmental and agronomic conditions.

Overall, the variability in melatonin effectiveness appears to arise from species-specific antioxidant capacity, differences in melatonin receptor sensitivity, and environmental influences such as soil chemistry. The interaction of these factors ultimately determines how efficiently plants absorb and utilize exogenous melatonin under aluminum stress.

### 1.9. Practical Applications and Implications

The incorporation of melatonin into agricultural practices represents a promising strategy for improving crop resistance, particularly in regions with acidic soils where aluminum toxicity is prevalent. One practical approach is the application of melatonin as a foliar spray or soil treatment, which can be integrated into routine fertilization and soil management programs during the growing season to help protect crops from aluminum stress [7]. Melatonin application may be especially effective during critical developmental stages, such as seedling establishment and root formation.

Furthermore, melatonin use can be combined with other soil management practices, including liming and the application of organic manure, to further alleviate soil acidity and enhance overall soil health [103].

Another promising approach is the development of melatonin-enriched formulations combined with nutrient bases, fertilizers, or bio-stimulants. These could be designed as slow-release systems to provide continuous protection against aluminum toxicity throughout the growing season. Melatonin-based seed coatings also represent an emerging technique for improving seedling vigor and stand establishment in acidic soils. Further research is needed to evaluate the effectiveness of melatonin under hydroponic or controlled environment systems, which would expand its applicability across different agricultural production methods [104]. Overall, these strategies have the potential not only to reduce aluminum toxicity but also to enhance crop growth, yield, and long-term soil fertility.

### 1.10. Advantages and Disadvantages of Melatonin in the Control of Soil Quality

Melatonin can play a significant role in soil management due to its multiple advantages. It reduces the effects of aluminum toxicity and promotes plant growth in acidic soils [105]. Melatonin also provides cytoprotective effects by scavenging free radicals that cause oxidative damage, thereby improving root development, nutrient uptake, and overall plant health [6]. In addition, melatonin may enhance soil microbial activity, which contributes to improved soil fertility and structure. Its low cost and ease of application make it an ideal option for farmers seeking a safe and effective means to mitigate aluminum toxicity without relying on conventional chemical treatments [106]. The effectiveness of melatonin can vary depending on soil type, crop species, and environmental conditions [105]. The optimal concentration and method of application require further investigation to ensure consistent results across different crops and soils. Additionally, the long-term effects of melatonin on soil ecosystems and plant health remain largely unknown [107]. Excessive use of melatonin, which may suppress its natural signaling functions, could potentially exacerbate soil acidity if underlying soil management issues are not addressed. Therefore, while melatonin offers promising benefits, it should be viewed as a complementary tool rather than a standalone solution; reliance on agrochemicals alone provides temporary fixes and cannot fully resolve global food security challenges [108].

Overall, the use of melatonin in agriculture may be more cost-effective than other strategies for alleviating aluminum stress, such as lime application or genetic modification of crops. Melatonin synthesis is relatively inexpensive, and its use has the potential to improve crop yield and quality in aluminum-affected regions, providing farmers with significant economic benefits. Moreover, if melatonin also enhances plant resistance to pests and diseases while promoting healthy growth, it could reduce the reliance on additional agricultural inputs such as pesticides and fertilizers, leading to more efficient crop management and lower production costs [109]. From an environmental perspective, melatonin offers a sustainable approach to mitigating soil acidity and reducing aluminum toxicity. Being a natural molecule, melatonin is environmentally friendly, non-toxic, and easily metabolized by soil microorganisms, unlike chemical soil amendments that can cause pollution [110]. Its application could help minimize the environmental impact associated with excessive use of fertilizers and soil conditioners. However, care must be taken to avoid negative effects on nontarget organisms or beneficial soil microbes. Additionally, large-scale agricultural use of melatonin raises considerations regarding its availability, sourcing, production, and distribution [103].

### 1.11. Future Directions and Research Gaps

Although recent studies suggest that melatonin can alleviate aluminum-induced stress in plants, several areas require further investigation to fully assess its agricultural potential. A key research focus should be on understanding the molecular mechanisms of melatonin action across different plant species and environmental conditions. Further studies on how melatonin interacts with other phytohormones and stress-signaling molecules could provide deeper insights into its functional roles and potential applications. Additionally, the effects of melatonin under varying soil types and climatic conditions need to be explored to establish standardized guidelines for its effective use in diverse agricultural systems [56]. A key focus for future research is determining the optimal method and dosage of melatonin application in crops. While existing studies show positive effects, it is still necessary to identify the most effective concentrations and timing for application. This knowledge could help prevent issues related to under- or over-application and ensure standardized use across different farming systems. Additionally, long-term studies are needed to assess the broader effects of melatonin on soil properties, microbial communities, and overall ecosystem balance [65].

Biotechnology offers promising opportunities to enhance melatonin synthesis in plants, enabling the development of crop varieties with greater tolerance to aluminum toxicity as well as other biotic and abiotic stresses. Since the genetic regulation of the melatonin biosynthesis pathway is well understood, scientists can genetically modify plants to produce naturally higher levels of melatonin. This approach could provide a sustainable solution by reducing the need for exogenous melatonin applications and allowing plants to better cope with stress factors in the soil environment independently [111]. The significant advances in CRISPR/Cas9 technology, combined with a deeper understanding of plant genomics, provide researchers with powerful tools to manipulate specific genes involved in melatonin biosynthesis. Upregulating melatonin biosynthetic genes or transferring melatonin-producing genes from other plants into crop species could enhance stress tolerance [66]. However, this line of research is still in its early stages and faces several challenges. These include understanding how increased melatonin levels affect normal plant growth and development, as well as addressing regulatory hurdles and public acceptance of genetically modified crops [68].

The long-term effects of melatonin on soil quality and crop productivity remain largely unexplored, representing an important research gap. While several studies have shown that melatonin can reduce aluminum stress in the short term, little is known about its impact on soil microbial communities, nutrient cycling, and the overall stability of the soil ecosystem across multiple growing seasons. Further research is needed to determine whether repeated applications of melatonin may alter soil chemistry, modify microbial populations, or lead to the development of resistance factors in plants or soil microorganisms [112]. Large-scale use of melatonin in agriculture will require a careful and coordinated approach by farmers, researchers, and other stakeholders to manage potential drawbacks and ensure its long-term sustainability. Future research should focus on collecting long-term data on the chemical, physical, and biological properties of the soil such as pH, organic matter content, and microbial activity, to fully understand the implications of melatonin application [111].

## 2. Conclusions

Melatonin has emerged as a promising regulator of plant tolerance to aluminum toxicity, a major constraint in acid soils that limits plant growth and agricultural productivity. Extensive studies demonstrate that melatonin alleviates aluminum stress primarily through plant-mediated mechanisms, including enhancement of antioxidant defense systems, protection against oxidative damage, promotion of root development, and improved nutrient uptake. Evidence across diverse plant species indicates that melatonin consistently improves physiological performance under aluminum stress conditions. Rather than directly modifying soil chemistry, melatonin contributes to improved plant performance in acid soils by regulating stress-responsive pathways and maintaining cellular homeostasis. However, its effectiveness may vary depending on plant species, soil properties, and environmental conditions. Future research should focus on optimizing application protocols’ dose, timing, and delivery methods, validating long-term effects under field conditions across diverse agroecosystems, and elucidating its role in rhizosphere processes and plant–microbe interactions. Additionally, genetic approaches aimed at enhancing endogenous melatonin biosynthesis offer a promising strategy for improving crop resilience to acidic soil conditions.

## Figures and Tables

**Table 1:** The effects of aluminum toxicity and the protective role of melatonin.

Aluminum Toxicity Effect	Melatonin Protective Role	References
Increased Al^3+^ uptake (apoplastic and symplastic entry into roots, root apex vulnerable)	Reduces Al^3+^ uptake by strengthening cell wall integrity and modifying pectin composition, limiting Al^3+^ binding and entry into root cells	[26,27]
Cell wall rigidification (binding to pectins, limits elongation)	Regulates cell-wall-modifying enzymes (pectin methylesterases, expansions), maintaining flexibility and promoting root elongation	[28]
Metabolic disruption (enzyme inhibition, phosphate transport affected, low ATP)	Stabilizes enzymes, preserves mitochondrial function, sustains ATP production under Al stress	[29]
Oxidative stress (ROS accumulation → membrane, protein, DNA damage)	Directly scavenges ROS; upregulates antioxidant enzymes (SOD, CAT, APX) to prevent oxidative damage	[30,31]
Cytoskeleton disruption (microtubules, actin filaments → impaired cytokinesis and elongation)	Stabilizes cytoskeleton, maintains cell shape, division, and elongation	[20]
Root apex damage (reduced meristem activity, decreased root length and surface area)	Promotes meristem activity, restores auxin transport, enhances root elongation and lateral root formation	[32]
Nutrient deficiency (competition with P, K, Ca, Mg → reduced absorption)	Regulates ion transporters, improves nutrient homeostasis and uptake	[18]
Hormonal disruption (auxin transport impaired → distorted root morphology)	Maintains hormonal balance, supports normal auxin distribution and healthier root architecture	[33]
Overall plant health (reduced growth, yield, stress resilience)	Multi-level protection: antioxidant defense, cell wall and cytoskeleton stabilization, energy metabolism maintenance, nutrient and hormone regulation → improves root growth, crop productivity, and resilience in acidic soils	[34]

**Table 2:** Growth effects of melatonin in plants.

Organism	Experimental Approach	Main Findings	References
*Phalaris canariensis*, *Triticum aestivum*, *Avena sativa*, *Hordeum vulgare*	Application of melatonin (10–8 to 10–4 µM) to coleoptile sections and post-germination seedlings to assess root growth	Enhanced coleoptile growth in *Phalaris* and *Hordeum* at 10–7 and 10–6 µM; mixed results in *Triticum* and *Avena*; increased root growth in *Triticum* at 10–8 to 10–6 µM, but inhibited at higher concentrations in *Phalaris* and *Avena*.	[42]
*Lupinus albus*	Addition of melatonin to de-rooted hypocotyls and hypocotyl sections	Hypocotyl growth was stimulated within the range of 10–8 to 10–5 µM melatonin.	[43]
*Lupinus albus*	Application of melatonin to de-rooted hypocotyls	Encouraged the formation of adventitious and lateral roots.	[44]
*Prunus cerasus* cv and various hybrids	Treatment of shoot-tip explants with 0.05–10µM melatonin	Rooting was promoted at lower melatonin concentrations, while higher concentrations led to inhibition, with variability across cultivars.	[45]
*Glycyrrhiza uralensis*	Comparison of melatonin levels in roots under different light conditions during plant development	Root melatonin levels were found to increase progressively with plant development over a 6-month period.	[46]
*Arabidopsis thaliana*	Exposure of 7-day-old seedlings to 50–500 µM melatonin in liquid culture	There was a significant increase in the number of lateral roots.	[47]
*Brassica juncea*	Addition of 0.1 or 100 µM melatonin to etiolated seedlings	Low concentration (0.1 µM) stimulated root growth, whereas a high concentration (100 µM) was inhibitory; the effect was most noticeable in 2-day-old seedlings.	[48]
*Mimosa pudica*	Treatment of cultured nodal segments with 100 µM melatonin or serotonin	Enhanced shoot multiplication and altered calcium ion levels.	[49]
*Oryza sativa*	Overexpression of melatonin biosynthesis gene AANAT	Root growth stimulation, increased seedling biomass, delayed flowering, and reduced grain yield.	[50]
*Solanum lycopersicum*	Overexpression of a melatonin catabolizing enzyme IDO	Resulted in reduced biomass and a decrease in the number of lateral leaflets.	[51]

**Table 3:** Different mechanisms of melatonin effects on plants.

Mechanism	Description	References
Antioxidant Activity	Melatonin scavenges reactive oxygen species (ROS) produced under aluminum stress.	[59]
Enhancement of Root Growth	Melatonin promotes root elongation and development, improving plant stability in acidic soils.	[60]
Modulation of Stress Response Pathways	Melatonin influences stress-related hormones and signaling pathways, reducing the impact of aluminum toxicity.	[61]
Improvement of Nutrient Uptake	Melatonin enhances nutrient absorption by mitigating aluminum’s inhibitory effects on root function.	[62]

**Table 4:** Summary of evidence for melatonin-mediated Al^3+^ tolerance mechanisms.

Mechanism Category	Crop/Species	Experimental System	Al^3+^ (AlCl_3_) Concentration	Melatonin Dose and Application	Key Response Parameters	Direct Al^3+^ Stress Evidence	References
**Cell wall modification**	Soybean (*Glycine max* L.)	Hydroponic	50–100 μM	50 μM, root application	Reduced Al deposition in cell wall, decreased pectin and hemicellulose 1 content, increased pectin methyl esterification, downregulated lignin synthesis, alleviated growth inhibition	Yes (cell wall Al fractions quantified; gene expression of cell wall-related genes)	[82]
	Wheat (*Triticum aestivum* L.)	Hydroponic	50 μM	20 μM, root application for 24 h	Decreased root tip Al content (19.0–15.5%), suppressed pectin methylesterase activity, altered cell wall polysaccharide composition	Yes (Al accumulation in root tips measured)	[10]
	Hickory (*Carya cathayensis*)	Hydroponic + pot (acid soil)	200 μM/pH 4.8 soil	100 μM, root application	Decreased cell wall pectin and hemicellulose, reduced Al-induced ROS, transcription factors CcC3H12 and CcAZF2 upregulated	Yes (cell wall component quantification; transcriptomic analysis)	[79]
**Organic acid exudation**	Soybean (*Glycine max* L.)	Hydroponic	50 μM	50 μM, root application for 24 h	Increased citrate and malate exudation, upregulated ALMT and MATE transporter expression, reduced Al-induced root growth inhibition	Yes (organic acids quantified in root exudates; gene expression analysis)	[5]
	Alfalfa (*Medicago sativa* L.)	Hydroponic + pot (acid soil	30 μM/pH 4.5 soil	100 μM, root application (hydro) or irrigation (pot)	Enhanced malate secretion, reduced Al accumulation in roots, transcriptomic reprogramming of organic acid metabolism genes	Yes (Al accumulation quantified; transcriptomic analysis)	[13]
**Antioxidant defense**	Strawberry (*Fragaria × ananassa* Duch.)	Pot (greenhouse)	100 μM	50–100 ppm, foliar spray	Upregulated SOD, CAT, APX, GR, GST, PAL activities; decreased H_2_O_2_ and MDA levels; enhanced growth, photosynthesis and fruit quality under Al stress	Yes (biochemical and physiological assays under Al stress)	[83]
	Maize (*Zea mays* L.)	Hydroponic	100 μM	50 μM, foliar spray	Increased shoot and root biomass, improved C and N metabolism, reestablished redox homeostasis via increased SOD, CAT, APX, GR activities	Yes (detailed antioxidant enzyme assays; ROS and MDA measurements)	[76]
	*Brassica napus* L.	Hydroponic	100 μM	100 μM, root application	Increased SOD, CAT, POD, APX activities; elevated proline, chlorophyll, anthocyanin; improved photosynthesis rate	Yes (multiparameter biochemical assays under Al stress)	[7,8]
	Alfalfa (*Medicago sativa* L.)	Hydroponic + pot (acid soil)	30 μM/pH 4.5 soil	100 μM, root application or irrigation (pot)	Reduced Al accumulation, decreased oxidative stress markers, improved root growth and biomass	Yes (Al content; ROS measurements; enzyme activity)	[13]
**Vacuolar sequestration**	Soybean (*Glycine max* L.)	Hydroponic	300 μM	1 μM, root application	Increased vacuolar Al sequestration, down-regulated GmCDT3, GmNrat1, GmIREG3, up-regulated GmALS1 transporter genes	Yes (vacuolar Al fraction quantification; gene expression analysis)	[82]
	Apple (*Malus hupehensis*)	Hydroponic	300 μM	1 μM, root application	Higher fresh and dry weight; increased photosynthetic capacity; more and longer roots; improved vacuolar H^+^/Al^3+^ exchange via MdSTOP1-MdNAC2-MdNHX2/ALS3 pathway	Yes (vacuolar Al compartmentalization demonstrated; molecular pathway elucidated)	[80]
	Rice (*Oryza sativa* L.)	Hydroponic	100 μM	20 μM, root application	Reduced Al accumulation in cell wall, enhanced vacuolar compartmentation via nitric oxide-dependent pathway	Yes (Al localization studied; NO pathway involvement shown)	[84,85]
**Hormonal regulation (auxin/GA/NO signaling)**	*Arabidopsis thaliana*	Hydroponic	50–100 μM	50–100 μM, root application for 48 h	Improved primary root elongation (~32%), restored root meristem and quiescent center activity, reduced NO production, altered cell cycle progression	Yes (root growth measurements; NO and cell cycle assays; quiescent center analysis)	[81]
**Combined mechanisms**	Hickory (*Carya cathayensis*)	Hydroponic + pot	200 μM	100 μM, root application	Reduced Al uptake, increased antioxidant activity, enhanced organic acid production, modulated transcription factors regulating cell wall genes	Yes (multiple endpoints; transcriptomic and molecular validation)	[79]

**Table 5:** Overview of experimental studies on melatonin alleviation of Al^3+^ toxicity in different crop species.

Crop Species	Cultivar/Line	Experimental System	Al^3+^ (AlCl_3_) Stress Condition	Melatonin Dose and Application Method	Main Observed Responses	Proposed Mechanisms	References
**Soybean (*Glycine max* L.)**	Not specified	Hydroponic	50 µM	50 µM, root application for 24 h	Root elongation ↑ 35%; Al accumulation in roots ↓ 40%; malate/citrate exudation ↑	Organic acid exudation; antioxidant enzyme activation	[5]
**Wheat (*Triticum aestivum* L.)**	Yangmai 12	Hydroponic	100 µM	20 µM, root application for 24 h	Root length ↑ 28%; Al content in root tips ↓ 50%; SOD, POD, CAT activities ↑ 30–60%	Cell wall modification; ROS scavenging; Al exclusion	[10]
**Maize (*Zea mays* L.)**	B73	Hydroponic	100 µM	50 µM, foliar spray every 2 days for 7 days	Shoot biomass ↑ 42%; root biomass ↑ 38%; H_2_O_2_ and MDA levels ↓ 45–55%; N and C metabolism improved	Redox homeostasis; enhanced nutrient assimilation	[76]
**Rice (*Oryza sativa* L.)**	Nipponbare	Hydroponic	100 µM	50 µM, root application for 72 h	Root elongation ↑ 30%; Al content in roots ↓ 35%; SOD, CAT activities ↑ 40%	ROS detoxification; Al exclusion	[85]
**Alfalfa (*Medicago sativa* L.)**	Zhongmu No. 1	Hydroponic + pot (acid soil)	30 µM/pH 4.5 soil	100 µM, root application or irrigation (pot)	Root growth improved; MDA ↓ 50%; Al content ↓ 35%; organic acid exudation ↑	Transcriptomic reprogramming; organic acid secretion	[13]
**Hickory (*Carya cathayensis* L.)**	Local variety	Hydroponic + pot (acid soil)	200 µM/pH 4.8 soil	100 µM, root application	Al uptake ↓ 45%; root and shoot biomass ↑ 30–40%; antioxidant enzymes ↑	Multi-pathway regulation (antioxidant + chelation)	[79]
**Tomato (*Solanum lycopersicum* L.)**	Micro-Tom	Hydroponic	100 µM	50 µM, root application	Root length ↑ 25%; NO signaling involved; reduced oxidative stress	NO-mediated antioxidant response	[12]
**Arabidopsis**	Col-0	Hydroponic	100 µM	50 µM, root application for 48 h	Primary root length ↑ 32%; Al-induced ROS ↓; cell death in root tip ↓	Interference with NO production; maintenance of meristem activity	[81]

Note: ↑ means increase and ↓ means decrease in observed responces.

## Data Availability

The data that support the findings of this study are available on request from the corresponding author. The data are not publicly available due to privacy or ethical restrictions.

## Abbreviations

Al^3+^, aluminum toxicity; ALMT, aluminum activated malate transporter; APX, ascorbate peroxidase; CAT, catalase; GA, gibberellin; GR, glutathione reductase; GST, glutathione S transferase; H_2_O_2_, hydrogen peroxide; HSP, heat shock protein; LEA, late embryogenesis abundant protein; MATE, multidrug and toxic compound extrusion transporter; MDA, malondialdehyde; NO, nitric oxide; PAL, phenylalanine ammonia lyase; PGPR, plant growth promoting rhizobacteria; PME, pectin methylesterase; POD, peroxidase; ROS, reactive oxygen species; RNS, reactive nitrogen species; SOD, superoxide dismutase; ABA, abscisic acid.
